# S60. SPANISH ADAPTATION AND VALIDATION OF THE SFRT-2 IN PATIENTS WITH SCHIZOPHRENIA AND HEALTHY CONTROLS

**DOI:** 10.1093/schbul/sby018.847

**Published:** 2018-04-01

**Authors:** Ainara Gómez-Gastiasoro, Javier Peña, Leire Zubiaurre-Elorza, Naroa Ibarretxe-Bilbao, Natalia Ojeda

**Affiliations:** University of Deusto

## Abstract

**Background:**

Social cognition (SC) impairment is common among patients with schizophrenia. This multidimensional construct comprises four domains: a) theory of mind (ToM); b) social perception (SP); c) attributional style (AS); and, d) emotion perception (EP). Especially within SC subdomains, SP, along with neurocognition, seems to be highly related to functional outcome in this population. However, nowadays and to our knowledge, only one measure of SP is available in Spanish and none of the existing SP measures have been adapted to native Spanish-speaking population. The scarce number of SP tests available, highlights the need of reliable instruments in Spanish. Therefore, the aim of the present study was to adapt and validate the SP assessment tool “Situational Feature Recognition Test 2” (SFRT-2) into native Spanish-speaking patients with schizophrenia and healthy controls (HC).

**Methods:**

The SFRT-2 was translated and retro-translated into Spanish. After that, one hundred and one patients with schizophrenia and 100 HC were assessed in order to obtain psychometric properties of the test. First, reliability of the SFRT-2 was studied with Cronbach’s alpha coefficients for actions hits, actions false positives, goals hits and goals false positives separately, in both patients and HC. Second, in patients’ group, concurrent validity was calculated using Spearman’s correlations in order to assess the relationship between SFRT hits and false positives scores and other SC measures such as ToM, EP, AS and global SC. Third, divergent validity was assessed in patients’ group by means of Spearman’s correlations in order to study the relationship between SFRT-2 and a neurocognition composite score. Finally, discriminant validity of SFRT-2 actions and objectives hits and false positives was obtained comparing schizophrenia and HC groups by means of Receiver Operating Characteristic (ROC) curve analysis. Percentiles for the SFRT-2 scores were also calculated and shown in order to facilitate clinical assessment of SP.

**Results:**

Regarding reliability of the test, internal consistency indexes of the SFRT-2 hits and false positives ranged from α = .66 to α = .90 in both groups, with higher indexes corresponding to patients’ group. Concerning convergent and divergent validity, SFRT-2 significantly correlated with other measures of SC, especially with ToM (SFRT-2 hits: r = .46, p < .01), and also, but to a lesser extent, with neurocognition composite score (SFRT-2 hits: r = .33, p < .01). Receiver Operating Characteristic (ROC) curve analysis showed that SFRT-2 hits and false positives discriminate well between patients with schizophrenia and HC, being false positives the indexes which best discriminated between both groups (actions false positives: AUC = .74, p < .001; objectives false positives: AUC = .78, p < .001).

**Discussion:**

Spanish adaptation and validation of the SFRT-2 showed good psychometric properties in both patients with schizophrenia and HC. In addition, reliability of the instrument seemed to be especially high among patients with schizophrenia. To our knowledge, this is the first adaptation and validation of an existing SP measure into native Spanish-speaking patients with schizophrenia. Given the good psychometric properties obtained by the Spanish adaptation, results further support the use of the SFRT-2 as an adequate measure to assess SP in patients with schizophrenia in both research and clinical practice. To that aim, SFRT-2 percentile scores for Spanish population were also provided in order to contribute to the appropriate detection of SP impairment in Spanish-speaking patients with schizophrenia.

